# Burden of unintentional drowning in China from 1990 to 2019 and exposure to water: findings from the Global Burden of Disease 2019 study

**DOI:** 10.1136/ip-2023-045089

**Published:** 2024-07-11

**Authors:** Ye Jin, Pengpeng Ye, Maoyi Tian, Leilei Duan, Amy E Peden, Richard Charles Franklin

**Affiliations:** 1 Division of Injury Prevention, National Center for Chronic and Non-communicable Disease Control and Prevention, Chinese Center for Disease Control and Prevention, Beijing, China; 2 The George Institute for Global Health, Newtown, New South Wales, Australia; 3 School of Public Health, Harbin Medical University, Harbin, Heilongjiang, China; 4 School of Population Health, University of New South Wales, Sydney, New South Wales, Australia; 5 College of Public Health, Medical and Veterinary Sciences, James Cook University, James Cook University, Queensland, Australia

**Keywords:** Mortality, Burden Of Disease, Risk/Determinants, Disability, Drowning

## Abstract

**Background:**

Drowning is an important contributor to the burden of deaths in China. Exposure to open water is a risk factor for drowning, but few studies quantify its impact on drowning. The purpose of this study was to provide an up-to-date analysis of unintentional drowning in China, including impact of exposure to open water.

**Methods:**

Chinese provincial data from the Global Burden of Disease Study 2019 were used to describe the burden of unintentional drowning in 33 provinces and changes from 1990 to 2019. Provincial outdoor open water resource data were used to explore the relationship between outdoor open water resources and drowning burden using K-median clustering analysis.

**Results:**

Between 1990 and 2019, the unintentional drowning incidence, mortality and disability adjusted life years (DALY) rates declined by 31.2%, 68.6% and 74.9%, respectively, with differences by age, sex and province. In 2019, the DALY rate for drowning was relatively higher in children under 20 year, the elderly over 80 years than other age groups and was relatively higher in men. There was no statistical difference in overall incidence rate by sex. Provincial differences in unintentional drowning burden show a positive relationship with the availability and size of outdoor open water.

**Conclusions:**

As expected availability of water increases drowning risk. There is a need to address drowning environmental risk especially among children and the elderly. Localised water safety plans which consider drowning burden and environmental risk factors are needed in China to ensure a sustained decline of unintentional drowning.

**WHAT IS ALREADY KNOWN ON THIS TOPIC**

- Drowning is an important cause of disease burden in China, especially for children. Several studies have explored the causes of drowning, but there is a lack of up-to-date national and provincial data identifying drowning burden and environmental risk factors.

**WHAT THIS STUDY ADDS**

- This study is the first one on the burden of drowning across all of China by province (excluding Taiwan). It comprehensively shows the national and provincial unintentional drowning burden in China in 2019 as well as changes in drowning burden from 1990 to 2019. The results reveal the high-risk drowning population and geographical differences of drowning burden, which would be expected to provide evidence for formulating targeted policies and strategies. This is the first study to show a link between exposure to water bodies and drowning incidence.

**HOW THIS STUDY MIGHT AFFECT RESEARCH, PRACTICE OR POLICY**

- This study shows the severity of drowning burden in China and provides preliminarily analysis of the environmental risk factors of drowning at the national and provincial level. Based on the results, this study can help to prioritise subnational water safety plans and activities and put forward targeted local drowning prevention strategies.

## Introduction

 Drowning is a significant cause of injury-related morbidity and mortality, yet remains an under recognised public health threat.^1 2^ China is a significant contributor to the global drowning burden. China, alongside India, Pakistan and Bangladesh, accounted for half the global incidence of fatal unintentional drowning in 2017.^1^

Studies exploring the epidemiology of drowning in China have largely focused on children, as drowning is the leading cause of death among children aged 1–14 years.^3^ However, national and provincial, all-age drowning literature is scarce in China, with the most recent analysis of Disease Surveillance Points System data reporting drowning nationally in China between 2006 and 2013.^4^

WHO in their drowning prevention implementation guide notes the need for better data as part of a broader strategy to address drowning.^5^ To aid in the development of evidence-informed drowning prevention strategies, there is a need for up-to-date analysis of drowning risk in China, stratified by age group, sex and region.^2^ Drowning risk is also heavily impacted by exposure to water. However, this issue has only had limited exploration globally and, outside of Taiwan, not previously been explored in China.^6 7^

To address these gaps in the literature, this study aims to provide up-to-date analysis of the unintentional drowning burden in China and its environmental risk—exposure to open water using the Global Burden of Disease (GBD) Study 2019 and outdoor open water resources data.^8^ In addition, the analysis incorporates data on the presence of outdoor open water (including precipitation, surface water resources and coastline to reflect outdoor open water resources) across various regions in China, to provide additional location-based environmental risk insights.

## Methods

In this study, we used the Chinese provincial data from the GBD Study 2019 to describe the burden of unintentional drowning in 33 provinces in China and changes from 1990 to 2019.^9^ Taiwan province is not included in this study due to limited access to data and its previous reporting in a 2017 study.^7^ The indicators that we used for estimating unintentional drowning burden were incidence (rate), death (rate) and disability-adjusted life years (DALY) presented with 95% uncertainty intervals. GBD specifies 13 external causes of injury (also known as E codes), which are mutually exclusive and collectively exhaustive based on International Classification of Diseases (ICD) codes.^3 8 10^ Drowning includes deaths and disability associated with unintentional immersion in water or another fluid.^11^ It is one of the injury external causes. The included ICD codes for drowning within ICD-10 are W65–W70.9, W73–W74.9; and for ICD-9, they are E910–E910.99. Drowning due to water transport or disasters (eg, flood) is assigned elsewhere in the GBD cause hierarchy and is, therefore, not included in this analysis.^1 11^ Injuries were categorised into 47 mutually exclusive and exhaustively collected injury classifications according to the S and T segment codes of ICD10 and 800–999 codes of ICD9.^1 9 11^ The methods of GBD for injury burden^1 3 8 12^ and drowning data have been published previously. All estimates followed the Guidelines for Accurate and Transparent Health Estimates Reporting^13^ (online supplemental appendix p 1–2). This study has been registered at Scientific Publications Team of the Institute for Health Metrics and Evaluation, University of Washington (ID: 1199-GBD2019-032020).

In order to understand the relationship between outdoor open water resources and drowning burden, we used indicators of precipitation, surface water resources and coastline to reflect outdoor open water resources. The key characteristic of each indicator of drowning burden is shown in the online supplemental appendix p 3 and outdoor open water resources are described below.

### Precipitation, surface water resources and coastline

In this study, precipitation refers to the depth of accumulated water (due to rain, snow or hail) (in millimetres), assuming no leakage, loss or evaporation, expressed as average annual precipitation. According to the actual observation values collected by rainfall observation points distributed in each region, the regional surface precipitation is analysed and calculated by using the isohyet line algorithm. The surface water resources (in Billion cubic metres) refers to the dynamic water volume of rivers, lakes, glaciers and other surface water bodies renewed year by year, that is, the runoff of local natural rivers. The amount of surface water resources in the regions is the natural annual runoff formed by local precipitation, excluding the inflow. We use provincial data of precipitation and surface water resources in 2019 from ‘2019 China Water Resources Bulletin’ formulated by the Ministry of Water Resources of China to describe outdoor inland open water resources.^14^

The coastline (in kilometres) is the dividing line and the connecting line between the sea and the land. It is generally divided into island coastline and continental coastline. The coastline for analysis in this study is continental coastline except for Hainan province since Hainan is an island with a total area of about 35 400 square kilometres. We use the data of the coastline from the local yearbook, local government websites and literature to describe outdoor coastal open water resources.^15^,^25^

Due to the lack of data sources, only the open water resources data of 31 provinces (excluding Hong Kong, Macao and Taiwan) are included in the cluster analysis.

### Analysis

STATA/IC V.15.0 (https://www.stata.com) was used to conduct a descriptive analysis of the incidence, mortality and DALY rates of drowning by sex and age group nationally in China and for its provinces. Combined with the relevant data of open water resources, precipitation and coastline in each province, the data of drowning incidence, mortality and DALY rate in each province are analysed by using the K-median clustering analysis method and taking Minkowski absolute distance as the measurement index.^26^

Three criteria are used as the grouping basis of cluster analysis (1) Calinski-Harabasz pseudo-F (CHF) criterion: the larger the CHF value, the more it supports the classification quantity; (2) within sum of squares (WSS) gravel map: find the inflection point from steep to gentle in the curve; (3) η^2^ coefficient: combined with WSS and CHF values, check if η^2^ coefficient is optimal or acceptable.^26^

Considering that some provinces are inland and have no coastline, this study divides 31 provinces in China into coastal and non-coastal provinces. Cluster analysis is conducted for each category to explore the relationship between open water resources and drowning burden.

### Patient and public involvement

No patient involved.

## Results

In 2019, an estimated 219 109 people drowned of which 56 524 people died from unintentional drowning in China. The drowned population included 92 064 men and 1 27 045 women, with 37 513 children less than 20 and 50 980 older people over 70 years old. Overall, there were approximately 2.9 million drowning deaths between 1990 and 2019 in China, with an average decrease of 3353 deaths per annum, Sichuan province (N=3 30 580; 11%) has the most proportion of deaths followed by Anhui (7%) and Hunan (7%) provinces.

### Change in drowning incidence, mortality and DALY rates at the national level, 1990–2019

**Figure 1:**
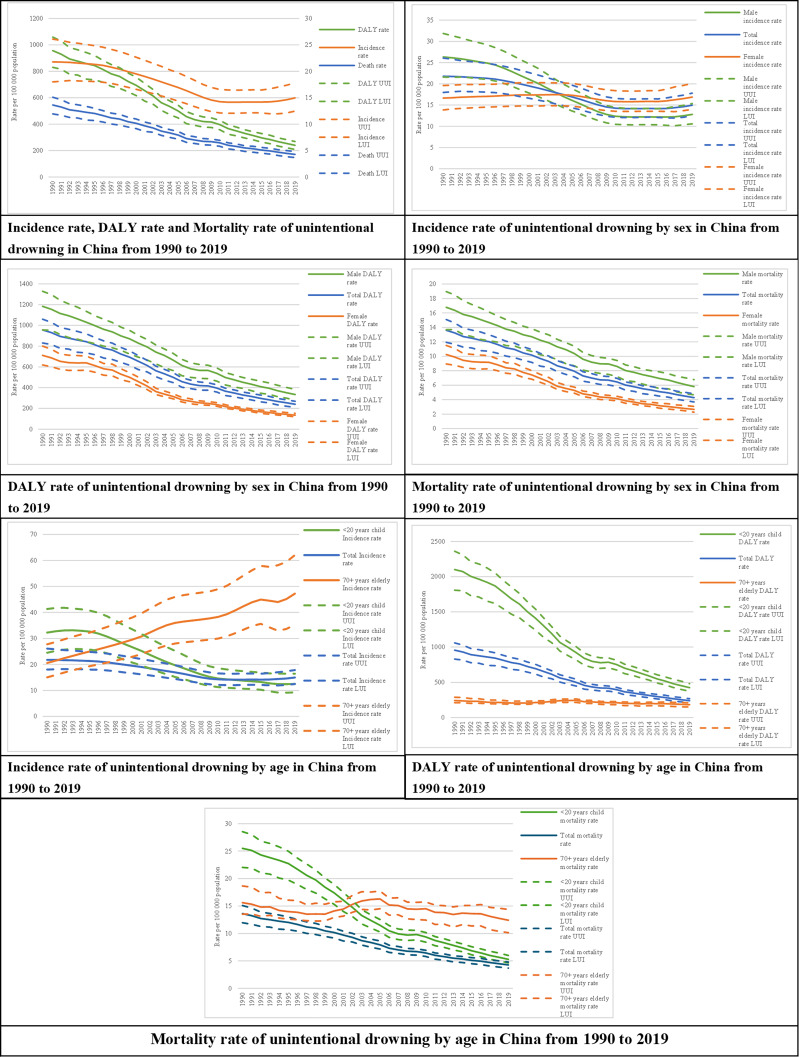
Incidence, DALY rate, mortality rate of unintentional drowning by sex and age group in China, 1990–2019. DALY, disability adjusted life years; UUI, Upper uncertainty interval; LUI, Lower uncertainty interval.

Between 1990 and 2019, the unintentional drowning incidence rate declined by 31.2% with a decrease between 1997 and 2010, preceded and followed by a levelling off in the 7 years before and after this period and an increase after 2017 (figure 1). The pattern change varied between men and women. The incidence rate decreased significantly in men while a slight increase with fluctuations was observed without significance in women (figure 1, online supplemental appendix p 11–12).

From 1990 to 2019, the mortality rate and DALY rate decreased by 68.6% and 74.9%, respectively. The patterns of change in mortality rate and DALY rate were similar between men and women (figure 1, online supplemental appendix p 11–12). The mortality rate, DALY rate and incidence rate all declined during the last 30 years in children less than 20 years. For elderly people aged 70 years and over, the incidence rate of drowning increased and the death rate and DALY rate remained steady (figure 1).

### Incidence rate, mortality rate and DALY rate of unintentional drowning at national level in 2019

The age-standardised cause-specific incidence, mortality and DALY rates for unintentional drowning are 15.0, 4.3 and 240.0 per 100 000, respectively.

**Figure 2:**
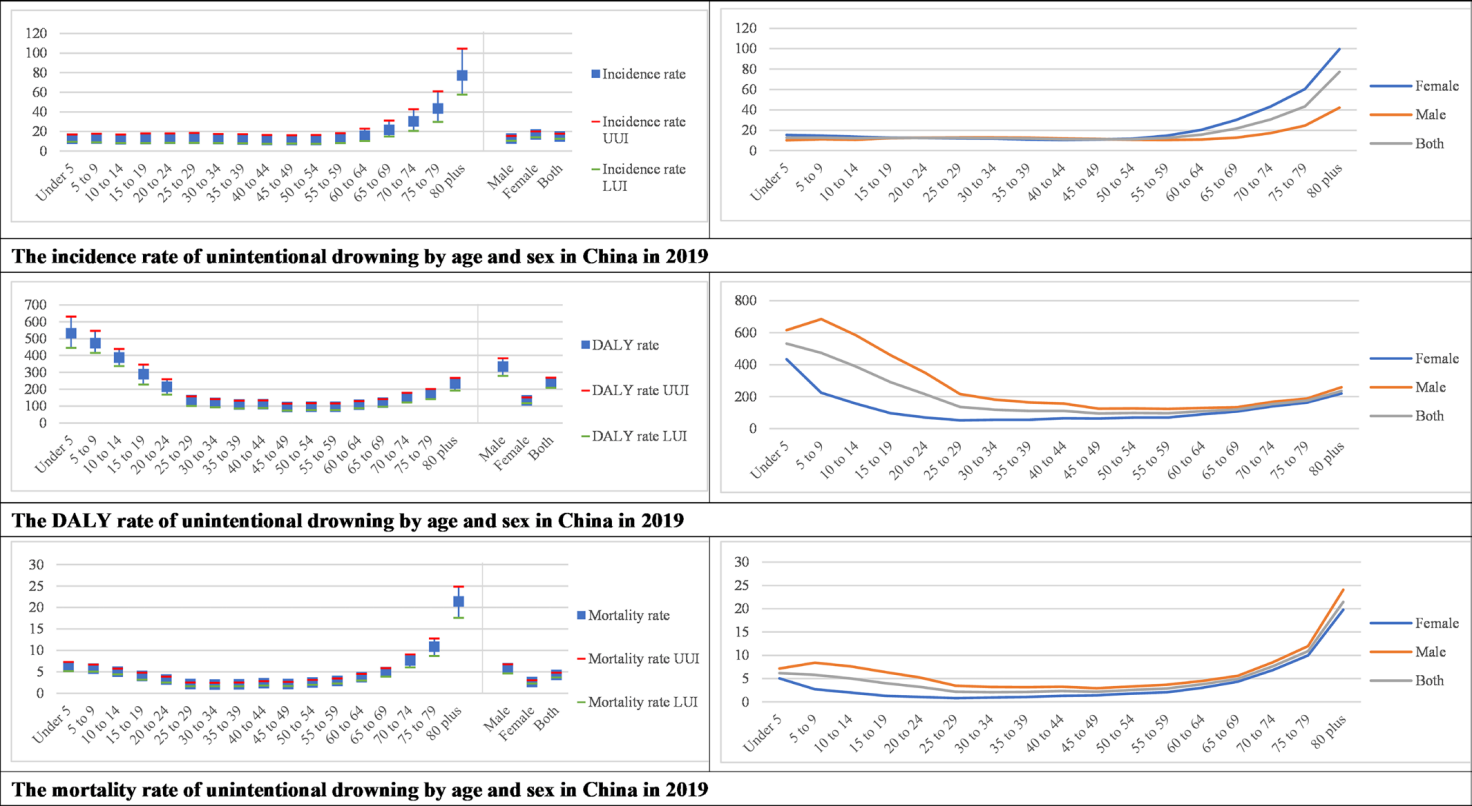
Incidence, DALY rate, mortality of unintentional drowning by age and sex in China in 2019. DALY, disability adjusted life years; UUI, Upper uncertainty interval; LUI, Lower uncertainty interval.

Both the unintentional drowning incidence rate and mortality rate increased with age (figure 2, online supplemental appendix p 6). The DALY rate among children under 20 and the elderly over 80 was higher than for other age groups. Children under 5 years of age had the highest DALY rate. Incidence of unintentional drowning in women is higher than that of men, although there is no statistical significance. The mortality rate and DALY rate of men were higher than that of women. The difference between men and women was greatest when comparing DALY rates, particularly among young people aged 5–30 years (figure 2, online supplemental appendix p 6–8).

### Change of incidence rate, mortality rate and DALY rate at subnational level during 1990–2019

Changes in incidence rate, mortality rate and DALY rate of unintentional drowning varied by province. Reductions in the unintentional drowning incidence rate were most apparent in Tibet, followed by Shanghai, Hebei, Macao and Tianjin. The incidence rate significantly increased in Inner Mongolia and Beijing (figure 4, online supplemental appendix p 11–12).

The mortality rate and DALY rate due to drowning decreased significantly in all regions. The reduction in mortality rate varied from 78.0% (Jiangxi) to 43.5% (Liaoning). The change in DALY rates varied from 82.2% (Shaanxi) to 45.8 (Liaoning) (figure 4, online supplemental appendix p 11–12).

### Incidence rate, mortality rate and DALY rate of unintentional drowning at subnational level in 2019

**Figure 3:**
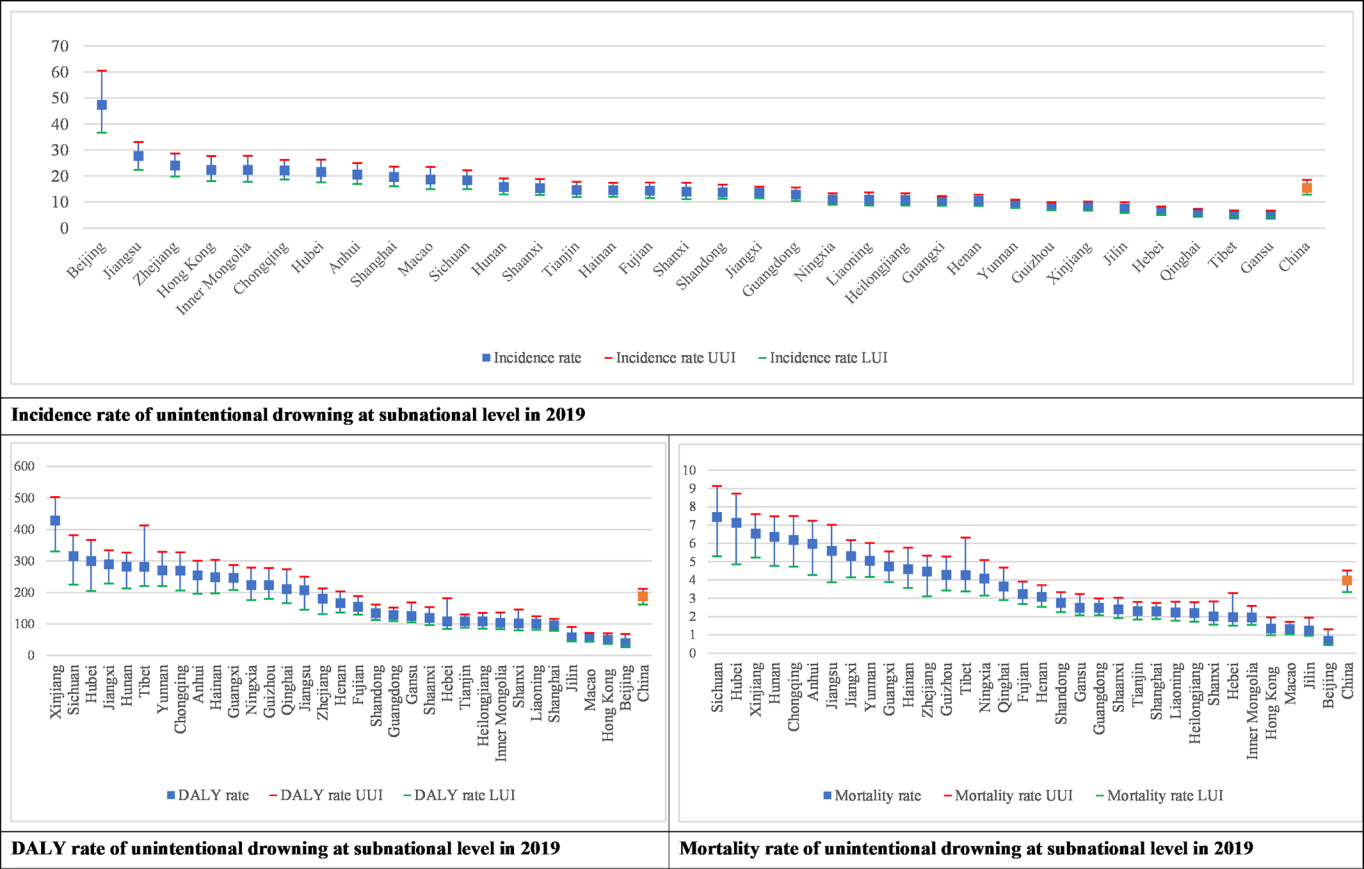
Incidence, DALY rate, mortality of unintentional drowning at subnational level in 2019. DALY, disability adjusted life years. UUI, Upper uncertainty interval. LUI, Lower uncertainty interval.

Beijing has the highest incidence of unintentional drowning, three times the national incidence, followed by Jiangsu and Zhejiang. The regions with the lowest incidence rate were Gansu, followed by Tibet and Qinghai (figures3  4, online supplemental appendix p 9–10).

**Figure 4:**
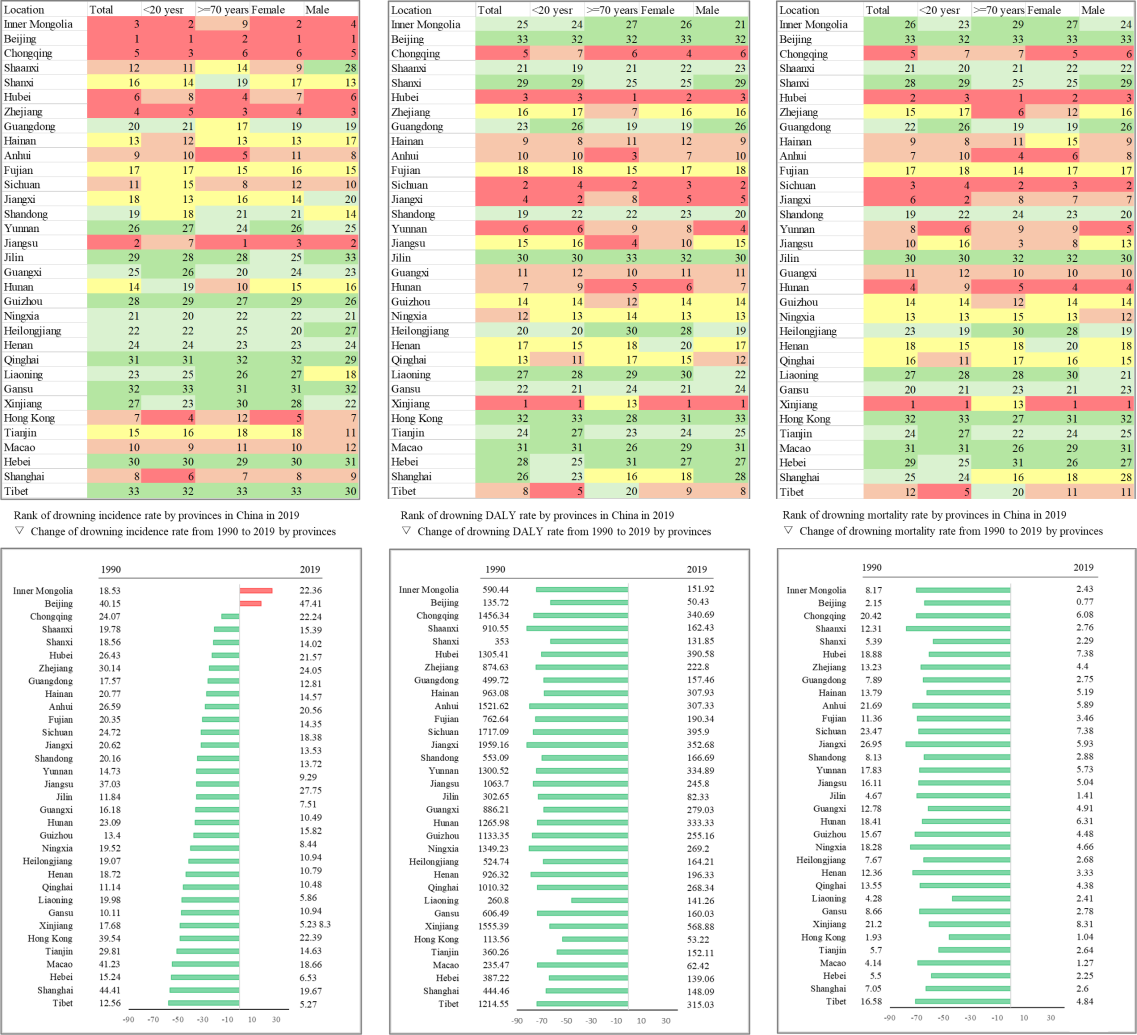
Rank of drowning incidence, DALYs and mortality rate by provinces in China in 2019 and change of drowning mortality rate from 1990 to 2019 by provinces. The upper three graphs are sorted from high to low based on the results, with rank 1 being the highest value and rank 33 being the lowest. Colours ranging from red, orange, yellow, light green and dark green are classified into five categories based on values. The three graphs at the bottom show the changes in drowning incidence, DALYs and mortality rates from 1990 to 2019. Green is a decrease; red is an increase. DALY, disability adjusted life years.

The high drowning mortality rates and DALY rates were seen in Sichuan, Hubei and Xinjiang. Low mortality rate and DALY rate are found in Macao, Jilin and Beijing (figure 3, online supplemental appendix p 9–10).

### Drowning-related environmental risk factors—precipitation, surface water, coastline

The subnational mortality rate, DALY rate, incidence rate and open water resources (precipitation, surface water, coastline) for 31 provinces are shown in online supplemental appendix p 13.

Cluster analysis results show that coastal provinces can be clustered into three categories while non-coastal provinces can be clustered into two categories.

The first category of coastal provinces includes Fujian and Guangdong. These provinces have more precipitation, surface water resources and longer coastline, but the incidence, death and DALY rate of drowning are relatively low. The second category of provinces includes Hebei, Liaoning, Shandong, Shanghai and Tianjin. They have less surface water resources, and lower death and DALY rates for drowning. But the drowning incidence rate and precipitation of Shanghai are relatively high and the coastlines of Liaoning and Shandong provinces are relatively long. For the third category, Jiangsu, Hainan, Guangxi and Zhejiang provinces tend to have high precipitation, surface water resources or long coastline. The drowning deaths and DALY rates are also relatively high. The incidence of drowning is relatively high in most provinces, except Guangxi Province (table 1).

**Table 1:** The characteristics of categories after cluster analysis and the ranking of provinces on mortality, DALY rate, incidence rate and precipitation, surface water and coastline in 2019^*^

Category		Province	Mortality rank	DALY rate rank	Incidence rate rank	Precipitation rank	Surface water source rank	Coastline rank
Provinces with coastlines	Group 1	Fujian	17	18	14	3	8	2
		Guangdong	21	20	18	1	5	1
	Group 2	Hebei	28	23	28	25	27	9
		Liaoning	25	28	20	18	22	5
		Shandong	19	19	16	21	24	3
		Tianjin	23	24	12	26	31	11
		Shanghai	24	29	8	8	28	10
	Group 3	Jiangsu	7	15	2	15	23	8
		Hainan	11	10	13	6	21	6
		Guangxi	10	11	22	5	3	7
		Zhejiang	12	16	3	2	10	4
Provinces without coastlines	Group 1	Henan	18	17	23	22	25	—
		Shanxi	27	27	15	24	26	—
		Heilongjiang	26	25	21	17	9	—
		Beijing	31	31	1	23	30	—
		Shaanxi	22	22	11	16	17	—
		Jilin	30	30	27	19	18	—
		Inner Mongolia	29	26	4	30	20	—
		Gansu	20	21	31	28	19	—
		Qinghai	16	14	29	27	12	—
		Ningxia	15	12	19	29	29	—
	Group 2	Hubei	2	3	6	14	14	—
		Xinjiang	3	1	26	31	13	—
		Xizang	14	6	30	20	1	—
		Guizhou	13	13	25	9	11	—
		Hunan	4	5	10	7	4	—
		Chongqing	5	8	5	10	15	—
		Yunnan	9	7	24	11	7	—
		Jiangxi	8	4	17	4	6	—
		Anhui	6	9	7	13	16	—
		Sichuan	1	2	9	12	2	—

* Colour coding: Quintile method is used to assign colour from dark to light according to the value from large to small.

DALY, disability adjusted life years.

For the non-coastal provinces, the first category includes Henan, Shanxi, Heilongjiang, Beijing, Shaanxi, Jilin, Inner Mongolia, Gansu, Qinghai and Ningxia. They have relative lower death rates and DALY rates and most provinces have less precipitation and surface water resources. Beijing and Inner Mongolia also have high incidence rate, but with less precipitation and surface water resources. The second category provinces are Hubei, Xinjiang, Xizang, Guizhou, Hunan, Chongqing, Yunnan, Jiangxi, Anhui and Sichuan. They have relative high death rate and DALY rate of drowning. They all have relative high precipitation or surface water resources. Except Xinjiang, Xizang, Guizhou, Yunnan and Jiangxi, most provinces in second category have a relatively high incidence rate of drowning (table 1).

## Discussion

The drowning incidence, death and DALY rates have generally shown a downward trend in the past 30 years, which is consistent with the global trend and in the Western Pacific region.^9^ This may be related to China’s infrastructure and education development in recent decades.^3^ The construction of relevant facilities, such as bridges and roads, and a national policy of improving drinking water and lavatories have resulted in reduced exposure to outdoor open water sources, which may be the reason for the reduction of drowning, although we also note that there has been an uptake of learn-to-swim programmes.^2 27^ Since hot weather can lead to higher risk of drowning,^28^ the popularisation of some cooling facilities in China over 30 years, such as air conditioning, may also have had an effect on drowning deaths by reducing the need for people to enter the water to cool down.^29 30^ In addition, the decline of child drowning may also be due in part to the development of education in China, the ‘one-child policy’ (1982–2016) enabling better supervision of children, or greater awareness of safety.^31^ The Ministry of Education’s annual communication to parents on child drowning risk and the need to strengthen strengthening parental care during holidays may also have contributed to a reduction in child drowning deaths,^32^,^34^ although we note a need for this to continue and be innovated to promote parents’ uptake of better supervision of children when on, in or near water.

Even with significant reductions in drowning burden among children and the elderly, there were high DALY rates in 2019 among these population. The DALY rate among children less than 20 years old is higher than the average same age drowning DALY rate globally and in the Western Pacific region.^9^ Despite the progress made in the past three decades, child drowning prevention in China still faces challenges. It is necessary to further strengthen children’s holiday care, enhance provision of swimming lessons and carry out safety and health education on drowning prevention and water activities for children and parents.^2^ The burden of elderly drowning is also relatively high. While the incidence of elderly drowning increased, the deaths and DALYs changed little over the 30 years. With an ageing population in China,^35^ drowning risk should also focus on elderly health and well-being initiatives. At present, the government has formulated a number of national and subnational policies to benefit the elderly, such as the national medium and long-term plans for actively responding to population ageing, but little attention has been paid to the problem of drowning among this at-risk age group.^36^

The female unintentional drowning incidence rate in China is similar to men. This is an unusual finding as men normally have higher rates than women. However, due to the lack of evidence, causal inference cannot be made, and more research should be conducted in this topic in the future.

### Need for local action

There are provincial differences in the burden of drowning in China, which our analysis has indicated is impacted by the amount of outdoor open water resources. On the whole, the burden of drowning is higher in areas rich in outdoor open water resources and high precipitation. Different provinces should explore the causes of drowning and carry out drowning prevention according to their own open water resources and drowning burden and compare initiatives across provinces to better understand what is working to reduce drowning.

The burden of drowning in the first category of coastal areas is low level considering the presence of abundant water resources; this may be related to better swimming ability and knowledge of water safety by residents in the area as well as a better rescue system and local government initiatives, such as joint efforts of multiple departments to carry out drowning prevention.^37 38^ These experiences and technologies can be extended to other areas with similar conditions, especially those with substantial water resources. At the same time, the region can further explore more efficient methods of drowning prevention by using advanced scientific and technological measures.

The burden of drowning in the second category is not high and there is lack of water resources in these regions. These provinces can carry out targeted drowning prevention in high-risk areas and among high-risk groups, such as drowning prevention in seaside water entertainment places in Liaoning and Shandong. The safety of limited water resources could be improved by strengthening open water supervision and reducing environmental risk factors. The third category has a relatively high burden of drowning, which is related to its rich open water resources. The local government and the community should prioritise drowning prevention efforts and learn from the successful experience from similar regions. Given the abundant water resources in these areas, policies should be focused on enlarging the area of safe water, including the seaside and swimming pool, and guarantee the safety in the safe water area by arranging enough lifeguards, providing lifesaving facilities and improving supervision by advance techniques (camera).^5 39^ At the same time, the government should strengthen advocacy to encourage more people to swim in safe areas while also promoting swimming techniques and water safety knowledge.

The drowning death and DALY rates of the first category comprising non-coastal provinces are low, which is related to the lack of abundant outdoor open water resources. Most provinces in this category have low incidence of drowning, except Beijing and Inner Mongolia. It is speculated that drowning occurring indoors in these provinces may be one of possible reasons of relative higher incidence of drowning; however, this needs to be explored further. The supervision of indoor water entertainment facilities and training of lifeguards should be strengthened as well as the management of water storage facilities.^40^ Investigation of new supervision camera techniques can also effectively improve indoor water safety.^39^ The second category has high death and DALY rate of drowning, which may be caused by rich water resources. However, five provinces in this category have lower incidence of drowning. This contradictory result suggests that drowning cases in these provinces are more likely to be fatal, which may be related to untimely rescue, low awareness of first aid or poor swimming ability. Prevention of fatal drowning requires correct rescue methods and fast first aid.^41^ Training the residents living near the open water will be beneficial for immediate and correct rescue. Further attention to drowning environmental risk reduction in these provinces is needed.

### Strengths and limitations

Limitations of GBD2019 data used in this study have previously been described.^8 10 42 43^ The reliability of incidence data could be improved since the main data source^44 45^ is limited national representative and there is a lack of data from 2006.^3^ In addition, there are some additional limitations specific to this study. Some data sources such as hospital surveillance systems may miss some non-fatal injury data arising from drowning incidents because some people may not go to hospital for care and be recorded as drowning case once they are rescued. Besides that, the misclassification of the death data could not be avoided entirely. Therefore, more survey data are needed as a supplement to existing data. This study only included unintentional drowning, excluding transport drowning, drowning caused by disasters and intentional drowning. It will cause an underestimation of the whole drowning burden and the data that is not included can be further analysed.^1 11^ Moreover, compared with the data of earlier years, the data estimated in recent years have more data sources and better quality. Earlier literature could be further adjusted for better data quality.^46 47^ This study conducts preliminary analysis on the impact of open water resources on risk of drowning. However, a further exploration of the direct causes of drowning is required, so as to provide more accurate evidence for local governments to adjust drowning prevention policies, this would include more detailed information about location and activity. Furthermore, precipitation, surface water source and coastline are indirect indicators to represent the risk of exposure to water. In addition to open water sources, there are some other outdoor environmental risk factors including temperature and geo-topography. There are also a range of other non-outdoor risk factors affecting drowning, such as indoor drowning environmental risk factors, swimming skills, alcohol consumption and lifejacket wear among many others.^7^ Further research is necessary to understand local drowning risk more precisely and provide more accurate evidence for provincial drowning prevention policies.

## Conclusion

In conclusion, this study shows a reduction in the unintentional burden of drowning in China from 1990 to 2019, while highlighting age, sex and provincial variations. The prevention of drowning in children and the elderly requires special attention. At present, China has no national drowning prevention plan. It is necessary to make such a plan to integrate multisectoral responses and enhance drowning prevention capacity. A higher authority is suggested to lead the plan formulation since multisectoral department-related drowning prevention, including health, education, public security, emergency management, maritime affairs and sports, needs to contribute to it. This study also explored the relationship between drowning and outdoor open water in different areas and puts forward corresponding suggestions according to the characteristics of each province. Provinces should design their own drowning prevention plans according to the characteristics of their own drowning burden. The whole country and provinces should also further explore the causes of drowning and formulate more targeted drowning prevention policies.

## Supplementary material

10.1136/ip-2023-045089 online supplemental appendix 1

## Data Availability

Data are available in a public, open access repository. Data are available upon reasonable request.
