# Supplementary material for: Burden of unintentional drowning in China from 1990 to 2019 and exposure to water: findings from the Global Burden of Disease 2019 study
Source: Inj Prev. 2024 Jul 11;31(5):e045089. doi: 10.1136/ip-2023-045089 (PMC12505047; doi:10.1136/ip-2023-045089)
Supplement: online supplemental appendix 1 [file ip-31-5-s001.pdf]

Appendix

1. GATHER Checklist

Item #	Checklist item Reported on	Reported on page #
**Objectives and funding**		
1	Define the indicator(s), populations (including age, sex, and geographic entities), and time period(s) for which estimates were made.	Main Pg. 2-4, Appendix Pg. 3
2	List the funding sources for the work.	Appendix Pg. 3
**Data Inputs**		
For all data inputs from multiple sources that are synthesised as part of the study:		
3	Describe how the data were identified and how the data were accessed.	Main Pg. 2-4, Appendix Pg. 3
4	Specify the inclusion and exclusion criteria. Identify all ad-hoc exclusions.	Main Pg. 2
5	Provide information on all included data sources and their main characteristics. For each data source used, report reference information or contact name/institution, population represented, data collection method, year(s) of data collection, sex and age range, diagnostic criteria or measurement method, and sample size, as relevant.	Main Pg. 2-4<br>http://ghdx.healthdata.org/gbd-2019/datainput-sources
6	Identify and describe any categories of input data that have potentially important biases (e.g., based on characteristics listed in item 5).	http://ghdx.healthdata.org/gbd-2019/datainput-sources
For data inputs that contribute to the analysis but were not synthesised as part of the study:		
7	Describe and give sources for any other data inputs.	1.Ministry of water resources of the people's Republic of China. 2019 China water resources bulletin. 2019.<br>2.Compilation Committee of Shanghai Yearbook. Shanghai yearbook 2020[M]. 2020. Shanghai: Shanghai Yearbook Agency<br>3.Guangdong Provincial Bureau of statistics and Guangdong Survey Corps of the National Bureau of Statistics. Guangdong statistical yearbook 2020[M]. 2020. Beijing: China Statistics Press<br>4.Hainan Provincial Bureau of statistics and Hainan Survey Corps of the National Bureau of Statistics. Hainan statistical yearbook 2020[M]. 2020. Beijing: China Statistics Press<br>5.Liaoning Provincial Bureau of statistics and Liaoning Survey Corps of the National Bureau of Statistics. Liaoning statistical yearbook 2020[M]. 2020. Beijing: China Statistics Press<br>6.Guangxi Statistics Bureau. Guangxi statistical yearbook 2020[M] . 2020. Beijing: China Statistics Press<br>7.Tianjin Provincial Bureau of statistics and Tianjin Survey Corps of the National Bureau of Statistics. Tianjin statistical yearbook 2020[M]. 2020. Beijing: China Statistics Press<br>8.Jiangsu Provincial Bureau of statistics and Jiangsu Survey Corps of the National Bureau of Statistics. Jiangsu statistical yearbook 2020[M]. 2020. Beijing: China Statistics Press<br>9.Shandong Provincial Bureau of statistics and Shandong Survey Corps of the National Bureau of Statistics. Shandong statistical yearbook 2020[M]. 2020. Beijing: China Statistics Press

		10.Zhejiang Provincial Bureau of statistics and Zhejiang Survey Corps of the National Bureau of Statistics. Zhejiang statistical yearbook 2020[M]. 2020. Beijing: China Statistics Press<br>11.Fujian Provincial Department of culture and Tourism. Reply on proposal No. 20221274 of the fifth session of the 12th CPPCC Provincial Committee. 2022. http://hyyyj.fujian.gov.cn/xxgk/zfxxgk/zfxxgkml/tyabl/202204/t20220406_5875496.htm (access Jul 25, 2022)<br>12.Hebei Provincial People's Government. Report of Hebei Provincial People's Government on the management of state-owned natural resource assets in the province in 2020. http://zrzy.hebei.gov.cn/heb/gongk/gkml/gggs/qtgg/xczx/10668449925936603136.html (access Jul 25, 2022)
For all data inputs:		
8	Provide all data inputs in a file format from which data can be efficiently extracted (e.g., a spreadsheet rather than a PDF), including all relevant metadata listed in item 5. For any data inputs that cannot be shared because of ethical or legal reasons, such as third-party ownership, provide a contact name or the name of the institution that retains the right to the data.	http://ghdx.healthdata.org/gbd-2019/datainput-sources<br>Appendix Pg. 4-13 Table 1-6
**Data analysis**		
9	Provide a conceptual overview of the data analysis method. A diagram may be helpful.	Main Pg. 2-4
10	Provide a detailed description of all steps of the analysis, including mathematical formulae. This description should cover, as relevant, data cleaning, data pre-processing, data adjustments and weighting of data sources, and mathematical or statistical model(s).	Main Pg. 2-4, Appendix Pg. 3
11	Describe how candidate models were evaluated and how the final model(s) were selected.	N/A
12	Provide the results of an evaluation of model performance, if done, as well as the results of any relevant sensitivity analysis.	N/A
13	Describe methods for calculating uncertainty of the estimates. State which sources of uncertainty were, and were not, accounted for in the uncertainty analysis.	Main Pg. 2-3, Appendix Pg. 3
14	State how analytic or statistical source code used to generate estimates can be accessed.	https://github.com/ihmeuw/ihme-modeling/tree/main/gbd_2019
**Results and Discussion**		
15	Provide published estimates in a file format from which data can be efficiently extracted.	http://ghdx.healthdata.org/gbd-results-tool<br>Appendix Pg. 4-13 Table 1-6
16	Report a quantitative measure of the uncertainty of the estimates (e.g. uncertainty intervals).	Main Pg. 5-6 and figures
17	Interpret results in light of existing evidence. If updating a previous set of estimates, describe the reasons for changes in estimates.	Main Pg. 5-6
18	Discuss limitations of the estimates. Include a discussion of any modelling assumptions or data limitations that affect interpretation of the estimates.	Main Pg. 8-9

2. Indicator of drowning burden

Incidence

The data sources of incidence are population-representative data to a certain extent and various research data, such as surveillance system, population survey, life registration data from published papers and unpublished datasets. Disease modelling software DisMod-MR V2.1 estimated the results. For those data with limited representative, the data will be adjusted using HAQ Index and World Health Survey new crosswalks. Data from New Zealand and Georgia inpatient data and the Vietnam National Injury Survey were also used for non-fatal estimation.[1,2,3]

Mortality

The data source of GBD 2019 is life registration data encoded by the ICD system or household mortality survey. GBD 2019 reclassifies the data with non-specific or unspecified codes and reduces noise. GBD 2019 also smooths the time trend and estimates the uncertainty interval for China and provincial and regional data. While analyzing cause of death data, oral autopsy site data was merged to accommodate small-scale oral autopsy studies, and updates were made when reassigning unclear cause of death codes.[1, 2, 3]

Disability adjusted life years (DALY)

DALY is an indicator for comprehensively measuring the health loss caused by diseases and injuries. It is the sum of years of life lost (YLLs) and years lived with disability (YLDs) caused by diseases and injuries. Disability weights used for estimating YLDs have been determined through analysis of data from population surveys and an open-access internet survey. In this methodological approach, participants are prompted to discern the comparative health status between randomly paired health states, each delineated by succinct descriptions of their key attributes.[1, 2, 3]

The method used to calculate the uncertainty interval of incidence, mortality and DALYs was reported in the GBD 2019 capstone paper.[1]

3. Role of the funding source

The funding source had no involvement in study design, data collection, data analysis, data interpretation, writing of the manuscript, and the decision to submit the manuscript for publication.

4. Data sharing

The authors had full access to all the data in the study and had final responsibility to submit for publication. The part of data is publicly available at Global Health Data Exchange (GHDx) online website (http://ghdx.healthdata.org/gbd-results-tool) and additional data could be requested from IHME.

References:

1. GBD 2019 Diseases and Injuries Collaborators. Global burden of 369 diseases and injuries in 204 countries and territories, 1990-2019: a systematic analysis for the Global Burden of Disease Study 2019. Lancet 2020; 396:1204–22. doi:10.1016/S0140-6736(20)30925-9

2. James SL, Castle CD, Dingels ZV, Fox JT, Hamilton EB, Liu Z, et al. Estimating global injuries morbidity and mortality: methods and data used in the Global Burden of Disease 2017 study. Inj Prev 2020; 26:i125–i53. doi: 10.1136/injuryprev-2019-043531

3. GBD 2019 Risk Factors Collaborators. Global burden of 87 risk factors in 204 countries and territories, 1990-2019: a systematic analysis for the Global Burden of Disease Study 2019. Lancet 2020; 396:1223–49. doi: 10.1016/S0140-6736(20)30752-2

Table 1 Age-standardized Incidence, DALY rate, Mortality rate of unintentional drowning in China by sex, 1990-2019

Year	Age-standardized Incidence rate per 100,000 (95% UI)			Age-standardized DALY rate per 100,000 (95% UI)			Age-standardized Mortality rate per 100,000 (95% UI)		
	Both	Male	Female	Both	Male	Female	Both	Male	Female
1990	21.78 (17.99, 26.07)	26.36 (21.61, 31.86)	16.64 (13.85, 19.60)	955.5 (829.81, 1058.59)	1183.42 (956.54, 1328.49)	710.62 (618.08, 799.51)	13.62 (11.94, 15.08)	16.79 (13.69, 18.96)	10.24 (8.95, 11.5)
1991	21.75 (18.13, 25.82)	26.16 (21.56, 31.36)	16.74 (14.07, 19.67)	929.61 (816.52, 1029.7)	1155.16 (952.15, 1296.56)	686.44 (600.94, 769.80)	13.24 (11.67, 14.62)	16.38 (13.70, 18.50)	9.89 (8.76, 11.10)
1992	21.67 (18.24, 25.50)	25.90 (21.58, 30.85)	16.83 (14.21, 19.81)	893.57 (778.93, 979.17)	1115.07 (912.60, 1242.95)	653.99 (577.57, 724.26)	12.73 (11.27, 13.90)	15.81 (13.02, 17.71)	9.44 (8.46, 10.40)
1993	21.57 (18.18, 25.31)	25.59 (21.47, 30.25)	16.92 (14.33, 19.84)	875.54 (770.88, 962.63)	1092.34 (882.39, 1206.80)	640.41 (565.81, 711.65)	12.50 (11.17, 13.64)	15.52 (12.74, 17.17)	9.27 (8.29, 10.27)
1994	21.45 (18.13, 25.10)	25.26 (21.25, 29.78)	17.00 (14.44, 19.85)	859.03 (741.21, 941.91)	1062.23 (866.20, 1175.19)	638.05 (566.38, 709.58)	12.24 (10.79, 13.37)	15.09 (12.35, 16.70)	9.18 (8.22, 10.17)
1995	21.31 (18.05, 24.85)	24.93 (21.08, 29.28)	17.05 (14.53, 19.89)	843.41 (735.27, 919.12)	1030.91 (844.08, 1135.35)	639.04 (567.80, 704.29)	12.02 (10.70, 13.00)	14.68 (12.16, 16.15)	9.18 (8.28, 10.07)
1996	21.11 (17.93, 24.57)	24.46 (20.70, 28.57)	17.12 (14.61, 19.93)	813.36 (717.19, 882.74)	997.77 (823.49, 1092.91)	611.77 (548.77, 670.80)	11.63 (10.42, 12.55)	14.25 (11.96, 15.60)	8.82 (7.99, 9.66)
1997	20.79 (17.72, 24.16)	23.74 (20.14, 27.74)	17.22 (14.67, 20.02)	781.12 (685.53, 850.89)	962.84 (800.08, 1059.96)	581.82 (520.59, 633.25)	11.19 (10.03, 12.09)	13.77 (11.47, 15.10)	8.41 (7.67, 9.09)
1998	20.39 (17.38, 23.73)	22.87 (19.41, 26.75)	17.32 (14.74, 20.15)	761.50 (671.52, 827.88)	936.97 (783.99, 1026.77)	568.49 (513.15, 625.76)	10.96 (9.82, 11.83)	13.47 (11.43, 14.69)	8.26 (7.53, 9.03)
1999	19.95 (16.97, 23.21)	21.94 (18.58, 25.68)	17.38 (14.79, 20.27)	723.08 (644.51, 786.67)	898.36 (760.18, 983.29)	529.49 (477.73, 582.52)	10.48 (9.47, 11.31)	12.99 (11.03, 14.18)	7.77 (7.08, 8.48)
2000	19.49 (16.60, 22.66)	21.06 (17.83, 24.72)	17.39 (14.79, 20.26)	693.70 (615.56, 754.30)	870.02 (722.78, 951.32)	498.49 (455.54, 543.09)	10.17 (9.17, 10.96)	12.70 (10.69, 13.84)	7.43 (6.84, 8.04)
2001	19.02 (16.18, 22.08)	20.14 (17.05, 23.68)	17.38 (14.80, 20.28)	653.54 (574.67, 708.09)	829.22 (693.76, 903.25)	458.63 (419.39, 500.38)	9.70 (8.64, 10.42)	12.23 (10.23, 13.34)	6.95 (6.41, 7.50)
2002	18.51 (15.78, 21.51)	19.11 (16.17, 22.41)	17.42 (14.85, 20.25)	613.15 (551.47, 662.95)	791.98 (666.79, 863.65)	414.51 (381.09, 454.11)	9.27 (8.46, 10.03)	11.86 (9.96, 12.96)	6.47 (5.97, 7.07)
2003	17.99 (15.35, 20.93)	18.04 (15.28, 21.12)	17.46 (14.88, 20.24)	563.01 (504.88, 607.36)	743.36 (630.46, 811.05)	362.38 (333.42, 394.21)	8.70 (7.88, 9.39)	11.31 (9.56, 12.36)	5.86 (5.43, 6.39)
2004	17.46 (14.87, 20.29)	17.02 (14.38, 19.94)	17.44 (14.83, 20.20)	533.39 (472.41, 576.15)	709.67 (591.56, 772.02)	336.94 (310.89, 366.47)	8.35 (7.51, 8.98)	10.94 (9.00, 11.91)	5.55 (5.12, 6.00)
2005	16.93 (14.37, 19.70)	16.12 (13.60, 18.88)	17.32 (14.74, 20.13)	499.44 (447.63, 543.54)	665.23 (564.03, 730.57)	314.24 (291.66, 340.70)	7.93 (7.15, 8.62)	10.38 (8.70, 11.39)	5.27 (4.89, 5.73)
2006	16.35 (13.87, 19.03)	15.25 (12.87, 17.87)	17.06 (14.51, 19.80)	457.66 (403.83, 494.95)	613.05 (514.43, 673.04)	283.62 (261.75, 307.04)	7.30 (6.56, 7.87)	9.60 (8.10, 10.53)	4.80 (4.44, 5.19)
2007	15.69 (13.32, 18.27)	14.32 (12.11, 16.80)	16.70 (14.25, 19.39)	434.31 (391.00, 471.53)	582.22 (499.46, 639.00)	268.31 (244.95, 292.38)	7.00 (6.35, 7.61)	9.19 (7.84, 10.11)	4.62 (4.22, 5.04)
2008	15.06 (12.79, 17.50)	13.46 (11.39, 15.73)	16.33 (13.97, 18.92)	419.41 (377.45, 455.29)	566.74 (489.35, 622.53)	253.62 (235.54, 273.25)	6.76 (6.10, 7.29)	8.96 (7.63, 9.85)	4.37 (4.03, 4.70)
2009	14.55 (12.38, 16.92)	12.78 (10.82, 14.91)	16.01 (13.69, 18.59)	416.03 (374.51, 447.31)	565.47 (485.83, 615.34)	247.36 (229.88, 265.39)	6.70 (6.04, 7.20)	8.94 (7.51, 9.71)	4.27 (3.97, 4.61)
2010	14.26 (12.13, 16.56)	12.39 (10.47, 14.44)	15.84 (13.53, 18.41)	396.15 (358.06, 428.36)	537.3 (462.35, 589.61)	236.87 (220.22, 255.66)	6.45 (5.82, 6.95)	8.59 (7.20, 9.42)	4.14 (3.84, 4.47)
2011	14.17 (12.07, 16.46)	12.24 (10.39, 14.28)	15.79 (13.50, 18.32)	366.07 (323.47, 394.86)	497.61 (425.59, 545.81)	217.47 (200.43, 235.21)	6.01 (5.32, 6.46)	8.03 (6.63, 8.81)	3.83 (3.50, 4.20)
2012	14.15 (12.07, 16.43)	12.20 (10.36, 14.22)	15.80 (13.52, 18.30)	348.22 (310.88, 378.05)	475.70 (407.16, 521.38)	204.10 (189.98, 219.49)	5.78 (5.13, 6.25)	7.77 (6.49, 8.55)	3.65 (3.37, 3.95)
2013	14.18 (12.12, 16.47)	12.20 (10.38, 14.20)	15.84 (13.54, 18.34)	329.49 (295.45, 356.87)	452.27 (392.57, 498.03)	190.86 (176.04, 205.94)	5.51 (4.83, 5.92)	7.45 (6.14, 8.22)	3.44 (3.15, 3.75)
2014	14.20 (12.17, 16.48)	12.21 (10.38, 14.19)	15.86 (13.55, 18.39)	314.33 (281.83, 342.94)	432.24 (369.72, 479.34)	181.48 (167.85, 197.02)	5.33 (4.69, 5.82)	7.21 (5.86, 8.01)	3.34 (3.04, 3.64)
2015	14.17 (12.12, 16.46)	12.18 (10.35, 14.17)	15.84 (13.53, 18.37)	296.45 (264.01, 328.42)	410.65 (345.72, 462.83)	167.94 (153.13, 184.20)	5.10 (4.49, 5.60)	6.93 (5.54, 7.84)	3.15 (2.82, 3.51)
2016	14.22 (11.98, 16.67)	12.17 (10.23, 14.33)	15.98 (13.48, 18.76)	283.58 (252.39, 316.30)	393.61 (335.99, 445.07)	159.94 (143.82, 177.09)	4.94 (4.33, 5.45)	6.72 (5.52, 7.60)	3.05 (2.69, 3.42)
2017	14.38 (11.95, 17.03)	12.27 (10.17, 14.68)	16.25 (13.46, 19.30)	267.14 (234.02, 296.28)	370.81 (313.33, 422.15)	150.84 (136.13, 168.00)	4.69 (4.05, 5.17)	6.37 (5.09, 7.23)	2.90 (2.58, 3.28)

Year	Age-standardized Incidence rate per 100,000 (95% UI)			Age-standardized DALY rate per 100,000 (95% UI)			Age-standardized Mortality rate per 100,000 (95% UI)		
	Both	Male	Female	Both	Male	Female	Both	Male	Female
2018	14.62 (12.13, 17.36)	12.48 (10.36, 14.94)	16.52 (13.69, 19.65)	253.44 (221.46, 283.12)	351.97 (298.31, 402.18)	142.95 (127.75, 161.03)	4.48 (3.86, 5.01)	6.08 (4.83, 7.03)	2.78 (2.44, 3.18)
2019	14.98 (12.47, 17.82)	12.80 (10.59, 15.36)	16.88 (14.01, 20.11)	239.89 (208.93, 268.23)	334.19 (278.89, 383.82)	134.11 (118.23, 151.95)	4.28 (3.68, 4.81)	5.82 (4.67, 6.77)	2.64 (2.26, 3.07)

Table 2 Incidence, DALY rate, Mortality rate of unintentional drowning in China by age and sex in 2019

	Incidence rate per 100,000 (95% UI)			DALY rate per 100,000 (95% UI)			Mortality rate per 100,000 (95% UI)		
Age group	Both	Male	Female	Both	Male	Female	Both	Male	Female
Under 5	12.66 (9.40, 17.03)	10.34 (7.66, 13.91)	15.37 (11.42, 20.57)	531.56 (446.14, 630.85)	615.53 (500.78, 753.13)	433.62 (366.82, 507.53)	6.17 (5.17, 7.32)	7.14 (5.81, 8.74)	5.03 (4.25, 5.89)
5 to 9	12.82 (8.98, 17.72)	11.31 (7.95, 15.52)	14.59 (10.12, 20.16)	473.46 (414.86, 547.31)	684.97 (593.59, 803.75)	224.56 (201.22, 254.60)	5.79 (5.07, 6.69)	8.38 (7.26, 9.83)	2.74 (2.45, 3.11)
10 to 14	12.12 (8.12, 17.07)	10.81 (7.38, 15.10)	13.67 (9.04, 19.48)	388.23 (338.41, 439.71)	583.69 (495.28, 678.44)	156.58 (134.31, 180.70)	5.07 (4.42, 5.75)	7.64 (6.48, 8.88)	2.04 (1.75, 2.35)
15 to 19	12.41 (8.27, 18.03)	12.12 (8.10, 17.56)	12.73 (8.39, 18.61)	289.69 (227.83, 346.59)	458.79 (344.01, 561.97)	96.19 (80.66, 112.37)	4.05 (3.18, 4.84)	6.42 (4.81, 7.86)	1.33 (1.11, 1.55)
20 to 24	12.60 (8.53, 17.99)	12.78 (8.74, 17.99)	12.40 (8.17, 18.15)	215.46 (167.94, 260.33)	348.89 (253.63, 433.15)	69.52 (53.69, 86.66)	3.22 (2.50, 3.90)	5.23 (3.79, 6.49)	1.02 (0.79, 1.28)
25 to 29	12.47 (8.41, 18.45)	12.99 (9.07, 19.10)	11.93 (7.76, 17.83)	136.05 (101.99, 158.58)	216.17 (151.04, 258.98)	52.98 (39.33, 68.21)	2.18 (1.62, 2.55)	3.49 (2.42, 4.18)	0.83 (0.62, 1.08)
30 to 34	12.28 (8.18, 17.44)	12.90 (8.67, 18.21)	11.65 (7.68, 16.68)	119.30 (93.71, 141.10)	182.41 (131.17, 222.55)	54.70 (41.93, 70.87)	2.07 (1.63, 2.46)	3.19 (2.29, 3.90)	0.93 (0.70, 1.21)
35 to 39	11.91 (7.99, 17.10)	12.69 (8.57, 18.06)	11.10 (7.25, 16.36)	110.72 (86.47, 132.27)	163.64 (116.03, 200.46)	55.77 (43.18, 70.88)	2.10 (1.63, 2.52)	3.13 (2.21, 3.84)	1.04 (0.79, 1.33)
40 to 44	11.33 (7.48, 16.33)	12.08 (8.09, 17.10)	10.56 (6.84, 15.68)	111.08 (88.62, 134.13)	156.02 (113.97, 196.76)	64.22 (49.60, 81.46)	2.32 (1.85, 2.81)	3.29 (2.40, 4.15)	1.32 (1.01, 1.69)
45 to 49	11.23 (7.57, 16.24)	11.51 (7.88, 16.55)	10.94 (7.22, 16.03)	94.87 (73.95, 114.70)	125.42 (87.28, 161.12)	63.13 (48.48, 80.02)	2.20 (1.69, 2.67)	2.93 (2.03, 3.79)	1.44 (1.10, 1.86)
50 to 54	11.26 (7.34, 16.48)	10.86 (7.17, 15.81)	11.68 (7.51, 17.41)	97.54 (77.44, 118.28)	125.72 (87.62, 162.30)	69.09 (53.70, 86.99)	2.55 (2.02, 3.11)	3.31 (2.28, 4.28)	1.78 (1.37, 2.26)
55 to 59	12.56 (8.25, 18.30)	10.54 (7.05, 15.28)	14.59 (9.57, 21.47)	97.12 (77.41, 116.07)	123.90 (85.69, 158.22)	70.08 (54.34, 87.77)	2.89 (2.29, 3.48)	3.72 (2.54, 4.79)	2.05 (1.56, 2.60)
60 to 64	15.65 (10.39, 22.88)	10.88 (7.35, 15.69)	20.46 (13.53, 30.20)	109.43 (88.45, 130.04)	128.86 (89.63, 163.13)	89.81 (70.34, 112.41)	3.78 (3.02, 4.54)	4.49 (3.09, 5.72)	3.06 (2.37, 3.87)
65 to 69	21.56 (14.85, 31.21)	12.73 (8.64, 18.41)	30.07 (20.59, 43.69)	120.82 (96.66, 141.86)	134.31 (86.17, 168.37)	107.81 (85.75, 133.35)	4.95 (3.90, 5.88)	5.56 (3.49, 7.03)	4.36 (3.44, 5.45)
70 to 74	30.49 (20.60, 42.78)	17.08 (11.60, 24.36)	43.25 (29.35, 60.52)	153.51 (122.2, 179.35)	168.18 (105.3, 212.73)	139.55 (112.44, 170.40)	7.66 (6.03, 9.05)	8.52 (5.23, 10.82)	6.84 (5.44, 8.49)
75 to 79	43.50 (29.93, 61.09)	24.58 (17.13, 34.16)	60.53 (41.64, 84.12)	174.72 (141.63, 202.16)	187.66 (121.7, 231.95)	163.07 (130.64, 199.08)	10.91 (8.72, 12.75)	11.95 (7.63, 14.89)	9.98 (7.83, 12.39)
80 plus	77.33 (57.72, 104.55)	42.21 (31.35, 57.75)	99.71 (73.67, 135.37)	234.63 (193.01, 268.09)	259.14 (178.46, 305.73)	219.02 (177.33, 262.68)	21.44 (17.58, 24.88)	24.06 (16.23, 28.64)	19.76 (15.61, 24.04)
Age-standardized	14.98 (12.47, 17.82)	12.80 (10.59, 15.36)	16.88 (14.01, 20.11)	239.89 (208.93, 268.23)	334.19 (278.89, 383.82)	134.11 (118.23, 151.95)	4.28 (3.68, 4.81)	5.82 (4.67, 6.77)	2.64 (2.26, 3.07)

Table 3 Incidence, DALY rate, Mortality rate of unintentional drowning in China by age and sex in 2019

Year	<20 years (per 100,000 (95% UI))			70+ years (per 100,000 (95% UI))			Total (per 100,000 (95% UI))		
	Incidence rate	DALY rate	Mortality rate	Incidence rate	DALY rate	Mortality rate	Incidence rate	DALY rate	Mortality rate
1990	32.28 (24.50, 41.36)	2101.23 (1808.31, 2359.51)	25.49 (22.02, 28.55)	20.54 (15.02, 27.75)	241.94 (211.36, 288.93)	15.60 (13.53, 18.64)	21.78 (17.99, 26.07)	955.50 (829.81, 1058.59)	13.62 (11.94, 15.08)
1991	32.78 (25.21, 41.69)	2071.85 (1801.17, 2309.73)	25.12 (21.90, 28.02)	21.47 (15.93, 28.63)	237.38 (210.30, 284.40)	15.34 (13.54, 18.45)	21.75 (18.13, 25.82)	929.61 (816.52, 1029.70)	13.24 (11.67, 14.62)
1992	33.06 (25.70, 41.77)	2005.14 (1729.65, 2212.64)	24.31 (21.05, 26.83)	22.40 (16.85, 29.65)	228.70 (202.56, 268.56)	14.81 (13.13, 17.43)	21.67 (18.24, 25.50)	893.57 (778.93, 979.17)	12.73 (11.27, 13.90)
1993	33.12 (25.95, 41.54)	1964.16 (1710.81, 2178.95)	23.83 (20.80, 26.41)	23.32 (17.75, 30.68)	228.56 (204.83, 267.71)	14.84 (13.31, 17.47)	21.57 (18.18, 25.31)	875.54 (770.88, 962.63)	12.50 (11.17, 13.64)
1994	32.97 (25.92, 41.22)	1919.93 (1652.65, 2123.23)	23.33 (20.09, 25.78)	24.19 (18.56, 31.53)	218.82 (197.22, 250.55)	14.21 (12.81, 16.40)	21.45 (18.13, 25.10)	859.03 (741.21, 941.91)	12.24 (10.79, 13.37)
1995	32.62 (25.72, 40.70)	1868.23 (1618.61, 2053.63)	22.75 (19.72, 24.97)	25.03 (19.36, 32.26)	215.13 (194.73, 247.54)	13.98 (12.61, 16.07)	21.31 (18.05, 24.85)	843.41 (735.27, 919.12)	12.02 (10.70, 13.00)
1996	31.90 (25.20, 39.88)	1778.75 (1545.51, 1948.67)	21.70 (18.92, 23.74)	25.87 (20.02, 33.30)	212.63 (192.54, 244.42)	13.87 (12.50, 15.97)	21.11 (17.93, 24.57)	813.36 (717.19, 882.74)	11.63 (10.42, 12.55)
1997	30.75 (24.32, 38.54)	1684.58 (1466.45, 1854.76)	20.60 (18.00, 22.64)	26.74 (20.72, 34.43)	206.57 (189.31, 232.52)	13.52 (12.31, 15.25)	20.79 (17.72, 24.16)	781.12 (685.53, 850.89)	11.19 (10.03, 12.09)
1998	29.36 (23.13, 36.87)	1610.06 (1416.27, 1759.43)	19.74 (17.38, 21.54)	27.64 (21.46, 35.62)	206.61 (187.30, 234.70)	13.56 (12.25, 15.52)	20.39 (17.38, 23.73)	761.50 (671.52, 827.88)	10.96 (9.82, 11.83)
1999	27.92 (21.84, 35.05)	1500.08 (1323.85, 1639.85)	18.45 (16.30, 20.13)	28.59 (22.23, 36.91)	205.60 (187.60, 231.87)	13.52 (12.30, 15.33)	19.95 (16.97, 23.21)	723.08 (644.51, 786.67)	10.48 (9.47, 11.31)
2000	26.58 (20.70, 33.48)	1408.80 (1240.96, 1538.02)	17.38 (15.30, 18.94)	29.55 (23.00, 38.07)	213.79 (196.30, 237.75)	14.11 (12.95, 15.77)	19.49 (16.60, 22.66)	693.70 (615.56, 754.30)	10.17 (9.17, 10.96)
2001	25.24 (19.65, 31.79)	1299.82 (1136.55, 1413.51)	16.08 (14.00, 17.47)	30.76 (23.96, 39.54)	218.07 (198.70, 238.49)	14.46 (13.15, 15.86)	19.02 (16.18, 22.08)	653.54 (574.67, 708.09)	9.70 (8.64, 10.42)
2002	23.77 (18.48, 30.05)	1189.57 (1061.71, 1290.22)	14.75 (13.08, 16.02)	32.24 (25.20, 41.40)	229.95 (209.67, 253.47)	15.31 (13.86, 16.94)	18.51 (15.78, 21.51)	613.15 (551.47, 662.95)	9.27 (8.46, 10.03)
2003	22.28 (17.28, 28.34)	1067.62 (944.33, 1154.86)	13.28 (11.75, 14.34)	33.78 (26.29, 43.22)	238.54 (216.44, 261.37)	15.90 (14.39, 17.60)	17.99 (15.35, 20.93)	563.01 (504.88, 607.36)	8.70 (7.88, 9.39)
2004	20.88 (16.16, 26.63)	995.14 (878.98, 1075.77)	12.39 (10.98, 13.40)	35.10 (27.35, 44.96)	242.40 (214.86, 262.12)	16.19 (14.29, 17.55)	17.46 (14.87, 20.29)	533.39 (472.41, 576.15)	8.35 (7.51, 8.98)
2005	19.68 (15.19, 25.18)	920.85 (818.92, 1005.34)	11.48 (10.26, 12.53)	36.00 (28.05, 45.96)	242.58 (216.02, 264.40)	16.30 (14.50, 17.73)	16.93 (14.37, 19.70)	499.44 (447.63, 543.54)	7.93 (7.15, 8.62)
2006	18.53 (14.31, 23.61)	841.87 (744.01, 916.95)	10.49 (9.29, 11.43)	36.50 (28.50, 46.65)	225.78 (199.56, 244.03)	15.19 (13.33, 16.43)	16.35 (13.87, 19.03)	457.66 (403.83, 494.95)	7.30 (6.56, 7.87)
2007	17.32 (13.38, 21.99)	797.32 (711.29, 869.56)	9.93 (8.88, 10.83)	36.87 (28.85, 47.20)	222.72 (199.04, 243.43)	15.09 (13.38, 16.50)	15.69 (13.32, 18.27)	434.31 (391.00, 471.53)	7.00 (6.35, 7.61)
2008	16.17 (12.49, 20.46)	778.78 (699.50, 848.50)	9.70 (8.73, 10.58)	37.24 (29.21, 47.64)	212.86 (186.63, 230.42)	14.48 (12.67, 15.71)	15.06 (12.79, 17.50)	419.41 (377.45, 455.29)	6.76 (6.10, 7.29)
2009	15.23 (11.74, 19.31)	785.99 (706.31, 847.23)	9.80 (8.81, 10.56)	37.69 (29.55, 48.09)	210.43 (182.84, 228.12)	14.43 (12.54, 15.67)	14.55 (12.38, 16.92)	416.03 (374.51, 447.31)	6.70 (6.04, 7.20)
2010	14.62 (11.27, 18.54)	752.27 (674.48, 813.68)	9.38 (8.43, 10.16)	38.27 (29.97, 48.97)	210.31 (181.82, 227.92)	14.51 (12.43, 15.73)	14.26 (12.13, 16.56)	396.15 (358.06, 428.36)	6.45 (5.82, 6.95)
2011	14.27 (11.03, 18.08)	699.69 (622.93, 757.86)	8.72 (7.78, 9.43)	39.27 (30.80, 50.25)	200.76 (168.81, 220.23)	13.86 (11.65, 15.17)	14.17 (12.07, 16.46)	366.07 (323.47, 394.86)	6.01 (5.32, 6.46)
2012	14.00 (10.84, 17.69)	665.69 (598.31, 723.64)	8.29 (7.46, 9.02)	40.74 (31.98, 52.13)	199.96 (169.81, 217.70)	13.79 (11.70, 15.05)	14.15 (12.07, 16.43)	348.22 (310.88, 378.05)	5.78 (5.13, 6.25)
2013	13.77 (10.68, 17.40)	628.40 (562.66, 686.26)	7.83 (7.01, 8.54)	42.40 (33.40, 54.23)	195.27 (162.19, 214.50)	13.46 (11.14, 14.84)	14.18 (12.12, 16.47)	329.49 (295.45, 356.87)	5.51 (4.83, 5.92)
2014	13.53 (10.52, 17.16)	591.43 (532.83, 647.97)	7.37 (6.65, 8.07)	43.94 (34.76, 56.27)	198.24 (168.58, 218.19)	13.70 (11.61, 15.10)	14.20 (12.17, 16.48)	314.33 (281.83, 342.94)	5.33 (4.69, 5.82)
2015	13.25 (10.30, 16.86)	549.36 (490.62, 607.57)	6.85 (6.13, 7.57)	44.96 (35.59, 57.88)	197.06 (165.97, 218.22)	13.60 (11.37, 15.12)	14.17 (12.12, 16.46)	296.45 (264.01, 328.42)	5.10 (4.49, 5.60)
2016	12.76 (9.72, 16.58)	517.07 (463.45, 576.91)	6.45 (5.77, 7.19)	44.50 (34.27, 57.53)	196.90 (164.23, 220.55)	13.57 (11.36, 15.27)	14.22 (11.98, 16.67)	283.58 (252.39, 316.30)	4.94 (4.33, 5.45)
2017	12.42 (9.22, 16.47)	481.13 (425.73, 543.43)	6.00 (5.32, 6.77)	43.98 (32.95, 58.15)	191.16 (156.86, 214.77)	13.12 (10.70, 14.75)	14.38 (11.95, 17.03)	267.14 (234.02, 296.28)	4.69 (4.05, 5.17)
2018	12.40 (9.15, 16.42)	452.81 (399.08, 516.02)	5.65 (4.98, 6.41)	45.12 (33.66, 59.45)	186.36 (152.19, 212.62)	12.76 (10.37, 14.59)	14.62 (12.13, 17.36)	253.44 (221.46, 283.12)	4.48 (3.86, 5.01)

Year	<20 years (per 100,000 (95% UI))			70+ years (per 100,000 (95% UI))			Total (per 100,000 (95% UI))		
	Incidence rate	DALY rate	Mortality rate	Incidence rate	DALY rate	Mortality rate	Incidence rate	DALY rate	Mortality rate
2019	12.51 (9.25, 16.44)	423.13 (372.68, 480.27)	5.29 (4.64, 5.99)	47.22 (35.21, 62.05)	182.11 (149.73, 208.93)	12.42 (10.13, 14.37)	14.98 (12.47, 17.82)	239.89 (208.93, 268.23)	4.28 (3.68, 4.81)

Table 4 Incidence, DALY rate, Mortality rate of unintentional drowning at subnational level in 2019

Rank	Location	Age-standardized Incidence rate per 100,000 (95% UI)	Rank	Location	Age-standardized DALY rate per 100,000 (95% UI)	Rank	Location	Age-standardized Mortality rate per 100,000 (95% UI)
-	China	15.40 (12.77, 18.44)	-	China	187.83 (161.66, 211.52)	-	China	3.97 (3.33, 4.51)
1	Beijing	47.41 (36.71, 60.53)	1	Xinjiang	428.68 (330.43, 502.71)	1	Sichuan	7.44 (5.30, 9.13)
2	Jiangsu	27.75 (22.39, 33.08)	2	Sichuan	315.47 (225.04, 382.15)	2	Hubei	7.13 (4.86, 8.72)
3	Zhejiang	24.05 (19.83, 28.69)	3	Hubei	300.35 (205.23, 367.27)	3	Xinjiang	6.54 (5.24, 7.60)
4	Hong Kong	22.39 (18.02, 27.65)	4	Jiangxi	289.66 (228.87, 334.40)	4	Hunan	6.36 (4.77, 7.48)
5	Inner Mongolia	22.36 (17.80, 27.75)	5	Hunan	282.36 (213.03, 326.54)	5	Chongqing	6.19 (4.73, 7.50)
6	Chongqing	22.24 (18.68, 26.25)	6	Tibet	281.80 (220.56, 413.17)	6	Anhui	5.98 (4.28, 7.25)
7	Hubei	21.57 (17.65, 26.35)	7	Yunnan	270.65 (220.77, 329.34)	7	Jiangsu	5.59 (3.87, 7.03)
8	Anhui	20.56 (16.96, 25.00)	8	Chongqing	269.82 (206.74, 327.24)	8	Jiangxi	5.31 (4.14, 6.18)
9	Shanghai	19.67 (16.09, 23.64)	9	Anhui	255.13 (196.18, 300.79)	9	Yunnan	5.05 (4.17, 6.03)
10	Macao	18.66 (14.95, 23.54)	10	Hainan	249.00 (197.36, 303.93)	10	Guangxi	4.75 (3.89, 5.56)
11	Sichuan	18.38 (15.02, 22.16)	11	Guangxi	246.62 (208.07, 287.58)	11	Hainan	4.59 (3.57, 5.77)
12	Hunan	15.82 (12.90, 19.15)	12	Ningxia	223.95 (175.90, 279.34)	12	Zhejiang	4.46 (3.11, 5.33)
13	Shaanxi	15.39 (12.72, 18.91)	13	Guizhou	223.70 (180.05, 277.67)	13	Guizhou	4.28 (3.43, 5.28)
14	Tianjin	14.63 (11.96, 17.77)	14	Qinghai	210.91 (166.14, 273.81)	14	Tibet	4.28 (3.37, 6.32)
15	Hainan	14.57 (11.99, 17.39)	15	Jiangsu	206.97 (145.36, 250.24)	15	Ningxia	4.08 (3.15, 5.09)
16	Fujian	14.35 (11.60, 17.47)	16	Zhejiang	180.62 (131.58, 213.32)	16	Qinghai	3.65 (2.90, 4.69)
17	Shanxi	14.02 (11.12, 17.42)	17	Henan	166.74 (136.74, 203.35)	17	Fujian	3.23 (2.69, 3.91)
18	Shandong	13.72 (11.31, 16.67)	18	Fujian	154.61 (130.37, 189.01)	18	Henan	3.09 (2.53, 3.72)
19	Jiangxi	13.53 (11.49, 15.93)	19	Shandong	134.62 (112.79, 161.59)	19	Shandong	2.75 (2.25, 3.34)
20	Guangdong	12.81 (10.43, 15.60)	20	Guangdong	128.49 (108.87, 152.66)	20	Gansu	2.48 (2.07, 3.24)
21	Ningxia	10.94 (8.93, 13.34)	21	Gansu	125.87 (105.24, 168.41)	21	Guangdong	2.48 (2.08, 2.99)
22	Liaoning	10.94 (8.65, 13.71)	22	Shaanxi	119.56 (96.26, 153.43)	22	Shaanxi	2.39 (1.91, 3.03)
23	Heilongjiang	10.79 (8.69, 13.39)	23	Hebei	108.84 (84.48, 181.89)	23	Tianjin	2.30 (1.84, 2.80)
24	Guangxi	10.49 (8.64, 12.34)	24	Tianjin	108.58 (88.49, 130.44)	24	Shanghai	2.29 (1.87, 2.76)
25	Henan	10.48 (8.52, 12.84)	25	Heilongjiang	108.27 (85.10, 135.47)	25	Liaoning	2.22 (1.78, 2.81)
26	Yunnan	9.29 (7.77, 10.98)	26	Inner Mongolia	104.09 (83.90, 136.64)	26	Heilongjiang	2.20 (1.73, 2.79)
27	Guizhou	8.44 (7.00, 9.97)	27	Shanxi	101.60 (79.92, 146.26)	27	Shanxi	2.01 (1.56, 2.83)
28	Xinjiang	8.30 (6.71, 10.20)	28	Liaoning	100.79 (81.40, 124.99)	28	Hebei	1.97 (1.51, 3.28)

Rank	Location	Age-standardized Incidence rate per 100,000 (95% UI)	Rank	Location	Age-standardized DALY rate per 100,000 (95% UI)	Rank	Location	Age-standardized Mortality rate per 100,000 (95% UI)
29	Jilin	7.51 (5.78, 9.94)	29	Shanghai	96.08 (78.52, 116.33)	29	Inner Mongolia	1.95 (1.54, 2.58)
30	Hebei	6.53 (5.10, 8.32)	30	Jilin	58.33 (45.15, 90.75)	30	Hong Kong	1.34 (0.99, 1.96)
31	Qinghai	5.86 (4.74, 7.23)	31	Macao	56.28 (44.87, 71.99)	31	Macao	1.32 (1.02, 1.71)
32	Tibet	5.27 (4.26, 6.61)	32	Hong Kong	49.95 (37.13, 70.45)	32	Jilin	1.24 (0.96, 1.95)
33	Gansu	5.23 (4.21, 6.59)	33	Beijing	39.66 (31.44, 68.64)	33	Beijing	0.68 (0.51, 1.31)

Table 5 Change of Incidence, DALY rate, Mortality rate of unintentional drowning at subnational level, 1990-2019

Change of Incidence rate % (95% UI)			Change of DALY rate % (95% UI)			Change of Mortality rate % (95% UI)		
Rank	Location	Change	Rank	Location	Change	Rank	Location	Change
-	China	-31.23 (-36.85, -25.45)	-	China	-74.89 (-78.44, -70.57)	-	China	-68.56 (-73.03, -63.46)
1	Tibet	-57.80 (-61.37, -53.38)	1	Shaanxi	-82.16 (-87.04, -75.53)	1	Jiangxi	-78.00 (-81.67, -72.96)
2	Shanghai	-56.61 (-60.84, -50.56)	2	Jiangxi	-82.00 (-85.18, -77.95)	2	Shaanxi	-77.62 (-83.24, -70.19)
3	Hebei	-55.31 (-60.82, -47.85)	3	Ningxia	-80.05 (-85.52, -72.69)	3	Ningxia	-74.52 (-80.96, -66.00)
4	Macao	-54.53 (-59.44, -47.10)	4	Anhui	-79.80 (-84.30, -74.51)	4	Henan	-73.10 (-79.39, -64.57)
5	Tianjin	-50.77 (-55.29, -45.58)	5	Henan	-78.81 (-84.07, -71.35)	5	Anhui	-72.84 (-78.63, -65.82)
6	Hong Kong	-48.30 (-53.82, -41.29)	6	Guizhou	-77.49 (-83.06, -69.24)	6	Guizhou	-71.43 (-78.26, -61.41)
7	Xinjiang	-48.24 (-53.69, -42.06)	7	Sichuan	-76.94 (-81.97, -69.88)	7	Tibet	-70.82 (-79.22, -59.34)
8	Gansu	-47.14 (-53.59, -38.78)	8	Jiangsu	-76.89 (-82.11, -70.52)	8	Inner Mongolia	-70.31 (-78.76, -59.30)
9	Liaoning	-46.68 (-53.08, -40.19)	9	Chongqing	-76.61 (-82.25, -69.35)	9	Chongqing	-70.22 (-77.35, -61.39)
10	Qinghai	-45.64 (-51.24, -39.40)	10	Fujian	-75.04 (-80.54, -68.11)	10	Jilin	-69.84 (-77.03, -61.47)
11	Henan	-43.60 (-52.43, -33.21)	11	Zhejiang	-74.53 (-79.95, -67.63)	11	Fujian	-69.53 (-75.69, -62.19)
12	Heilongjiang	-41.18 (-47.86, -33.50)	12	Inner Mongolia	-74.27 (-81.78, -64.04)	12	Macao	-69.31 (-76.50, -60.19)
13	Ningxia	-39.73 (-47.54, -31.56)	13	Yunnan	-74.25 (-81.62, -62.21)	13	Jiangsu	-68.71 (-75.47, -60.23)
14	Guizhou	-37.16 (-44.57, -28.11)	14	Tibet	-74.06 (-82.26, -62.45)	14	Sichuan	-68.53 (-75.20, -59.47)
15	Hunan	-35.95 (-43.00, -26.91)	15	Hunan	-73.67 (-78.73, -66.90)	15	Yunnan	-67.88 (-76.43, -54.25)
16	Guangxi	-35.51 (-42.88, -25.94)	16	Gansu	-73.61 (-80.74, -64.95)	16	Gansu	-67.84 (-75.46, -58.02)
17	Jilin	-35.12 (-44.82, -22.01)	17	Macao	-73.49 (-79.35, -65.10)	17	Qinghai	-67.71 (-75.51, -56.48)
18	Jiangsu	-34.90 (-42.61, -25.78)	18	Qinghai	-73.44 (-80.50, -63.10)	18	Zhejiang	-66.73 (-73.89, -58.29)
19	Yunnan	-34.85 (-41.36, -26.54)	19	Jilin	-72.80 (-79.35, -64.26)	19	Hunan	-65.75 (-72.44, -57.98)
20	Shandong	-34.15 (-41.69, -25.90)	20	Hubei	-70.08 (-76.65, -61.64)	20	Guangdong	-65.13 (-72.41, -55.42)
21	Jiangxi	-31.39 (-38.90, -21.49)	21	Shandong	-69.86 (-76.52, -62.13)	21	Heilongjiang	-65.00 (-73.57, -54.03)
22	Sichuan	-31.16 (-40.12, -22.03)	22	Heilongjiang	-68.71 (-76.66, -58.22)	22	Shandong	-64.60 (-71.96, -55.31)
23	Fujian	-30.19 (-39.11, -20.29)	23	Guangxi	-68.51 (-74.71, -61.05)	23	Beijing	-64.22 (-73.39, -52.06)
24	Anhui	-27.99 (-36.45, -18.87)	24	Guangdong	-68.49 (-75.57, -58.95)	24	Shanghai	-63.20 (-70.69, -53.12)
25	Hainan	-26.64 (-35.26, -15.55)	25	Hainan	-68.03 (-75.66, -57.53)	25	Hainan	-62.40 (-71.57, -50.52)
26	Guangdong	-25.75 (-34.14, -17.15)	26	Shanghai	-66.68 (-74.94, -55.28)	26	Guangxi	-61.55 (-68.82, -53.00)
27	Zhejiang	-24.70 (-33.89, -15.23)	27	Hebei	-64.09 (-73.58, -52.97)	27	Hubei	-60.91 (-69.44, -50.70)
28	Hubei	-22.19 (-31.65, -12.25)	28	Xinjiang	-63.43 (-71.12, -53.02)	28	Xinjiang	-60.78 (-68.60, -50.72)

Change of Incidence rate % (95% UI)			Change of DALY rate % (95% UI)			Change of Mortality rate % (95% UI)		
Rank	Location	Change	Rank	Location	Change	Rank	Location	Change
29	Shanxi	-21.14 (-31.15, -9.96)	29	Beijing	-62.84 (-72.55, -50.06)	29	Hebei	-59.01 (-69.42, -46.65)
30	Shaanxi	-20.27 (-29.52, -9.16)	30	Shanxi	-62.65 (-73.19, -49.85)	30	Shanxi	-57.59 (-68.72, -43.85)
31	Chongqing	-14.43 (-23.68, -3.44)	31	Tianjin	-57.78 (-68.55, -44.58)	31	Tianjin	-53.66 (-64.79, -40.72)
32	Beijing	17.59 (5.61, 33.79)	32	Hong Kong	-53.14 (-63.97, -38.82)	32	Hong Kong	-45.95 (-59.18, -28.32)
33	Inner Mongolia	26.34 (11.03, 43.51)	33	Liaoning	-45.84 (-62.35, -25.25)	33	Liaoning	-43.55 (-59.97, -23.71)

Table 6. The subnational mortality rate, DALY rate, incidence rate and related environmental risk factors - precipitation, surface water, coastline in 2019*

Province	Mortality rate (per 10,000)	DALY rate (per 10,000)	Incidence rate (per 10,000)	Precipitation (mm)	Surface water Source (Billion cubic meters)	Coastline (km)
Anhui	5.98	255.13	20.56	935.8	482.1	-
Beijing	0.68	39.66	47.41	506	8.6	-
Fujian	3.23	154.61	14.35	1730.7	1362.5	3752
Gansu	2.48	125.87	5.23	362.1	312.2	-
Guangdong	2.48	128.49	12.81	1993.6	2058.3	4114.4
Guangxi	4.75	246.62	10.49	1602.7	2103.8	1629
Guizhou	4.28	223.7	8.44	1246.1	1117	-
Hainan	4.59	249	14.57	1594.4	249.3	1944
Hebei	1.97	108.84	6.53	442.7	51.4	487
Henan	3.09	166.74	10.48	529.1	105.8	-
Heilongjiang	2.2	108.27	10.79	728.3	1305.7	-
Hubei	7.13	300.36	21.57	893.5	583.4	-
Hunan	6.36	282.36	15.82	1498.5	2091.2	-
Jilin	1.24	58.33	7.51	679.3	437.4	-
Jiangsu	5.59	206.97	27.75	798.5	163	954
Jiangxi	5.31	289.66	13.53	1710	2032.7	-
Liaoning	2.22	100.79	10.94	687.2	211.5	2110
Inner Mongolia	1.95	104.09	22.36	279.5	305.8	-
Ningxia	4.08	223.95	10.94	345.7	10.3	-
Qinghai	3.65	210.91	5.86	374	898.2	-
Shandong	2.75	134.62	13.72	558.9	119.7	3345
Shanxi	2.01	101.6	14.02	458.1	58.5	-
Shaanxi	2.39	119.56	15.39	759.4	469.7	-
Shanghai	2.29	96.08	19.67	1389.2	40.9	213
Sichuan	7.44	315.47	18.38	953.2	2747.7	-
Tianjin	2.3	108.58	14.63	436.2	5.1	153
Xizang	4.28	281.8	5.27	596.3	4496.9	-
Xinjiang	6.54	428.68	8.3	174.7	829.7	-
Yunnan	5.05	270.65	9.29	1008	1533.8	-
Zhejiang	4.46	180.62	24.05	1950.3	1303	2218
Chongqing	6.19	269.82	22.24	1106.8	498.1	-

* Color Coding: Quintile method is used to assign color from dark to light according to the value from large to small
